# Psychological distress, resilience, and coping among Ukrainian war refugees: cross-national differences across seven European host countries and Ukraine

**DOI:** 10.3389/fpubh.2026.1771440

**Published:** 2026-02-26

**Authors:** Małgorzata Szkup, Sabina Krsnik, Marta Kozybska, Elena Rousou, Olha Fedortsiv, Emilia Burbela, Sanja Stanisavljević, Agnieszka Kleszcz, Rita Fernandes, Marzena Mikla, Panagiota Ellina, Linda Czeponis, Tânia Lourenço, Karmen Erjavec

**Affiliations:** 1 Subdepartment of Social Nursing, Pomeranian Medical University in Szczecin, Szczecin, Poland; 2 Faculty of Health Sciences, University of Novo Mesto, Novo Mesto, Slovenia; 3 Subdepartment of Medical Law, Pomeranian Medical University in Szczecin, Szczecin, Poland; 4 Department of Nursing, Faculty of Health Sciences, Cyprus University of Technology, Limassol, Cyprus; 5 Ivan Horbachevsky Ternopil National Medical University of the Ministry of Health of Ukraine, Ternopil, Ukraine; 6 The College of Health Sciences, Academy for Applied Studies Belgrade, Belgrade, Serbia; 7 Eberswalde University for Health Professions, Eberswald, Germany; 8 RISE-Health, Nursing School, University of Porto, Porto, Portugal; 9 Department of Nursing, University of Murcia, Pascual Parrilla Murcian Institute of Biomedical Research (IMIB), Murcia, Spain; 10 RISE-Health, Nursing School of São José de Cluny, Funchal, Portugal

**Keywords:** coping strategies, cross-national comparison, mental health, psychological distress, public health, resilience, Ukrainian refugees

## Abstract

**Introduction:**

Armed conflict and forced displacement pose substantial risks to mental health, yet psychological outcomes among refugees vary considerably across national contexts. Understanding cross-national differences in distress, resilience, and coping is essential for designing effective public health responses to large-scale displacement crises such as the war in Ukraine. This study aimed to identify and compare cross-national differences in psychological distress, resilience, and coping strategies among Ukrainian war refugees residing in seven European host countries and internally displaced persons in Ukraine.

**Methods:**

A cross-sectional online survey was conducted among 1,631 Ukrainian adults residing in Poland, Germany, Spain, Cyprus, Slovenia, Portugal, Serbia, or displaced within Ukraine. Psychological distress was assessed using the Refugee Health Screener (RHS-15), the Hopkins Symptom Checklist (HSCL-25), and a DSM-5–based trauma symptom checklist. Resilience was measured with the Resilience Scale (RS-25), and coping strategies were assessed using the COPE Inventory (COPE-60). One-way analyses of variance (ANOVA) were used to examine cross-national differences in distress, resilience, and adaptive and maladaptive coping.

**Results:**

Significant cross-national differences were observed across all mental health indicators (*p* < 0.05). The highest levels of psychological distress were reported by refugees in Slovenia, Ukraine, Portugal, Spain, and Germany, whereas the lowest distress levels were observed in Cyprus and Poland. Resilience differed significantly between countries (*F* = 22.01, *p* < 0.001), with the highest mean levels reported in Poland and Germany and the lowest in Spain. Adaptive coping strategies were most frequently used in Poland, Germany, and Slovenia and least frequently in Cyprus and Serbia (*F*_(7, 1622)_ = 25.85, *p* < 0.001). Differences in maladaptive coping were also significant but less pronounced across countries.

**Conclusions:**

The mental health, resilience, and coping strategies of Ukrainian refugees vary substantially across host-country contexts. These findings underscore the importance of institutional, social, and policy environments in shaping psychological adaptation during forced displacement. Public health responses to refugee crises should address not only individual-level coping and resilience but also structural factors such as access to services, stability of reception systems, and integration support to reduce psychological distress and promote mental wellbeing.

## 1 Introduction

Mental health and its associated wellbeing constitute central elements of individual functioning and are key determinants of quality of life. In health policy practice, however, mental health is often reduced to the absence of a diagnosed disorder, which diminishes its broader meaning. The holistic approach promoted by the World Health Organization (WHO) emphasizes that health encompasses interconnected psychological, physical, and social dimensions, and that wellbeing cannot be equated solely with the absence of pathology. Contemporary global challenges—including dynamic social change, economic instability, climate-related stressors, and, above all, armed conflicts—have further underscored the complexity of mental health and its determinants. Armed conflicts exert particularly profound psychological effects, generating both direct trauma among affected populations and wider feelings of insecurity within societies (1, 2).

The war between Ukraine and Russia, which began in 2014 and escalated into full-scale armed conflict in 2022, has created one of the largest humanitarian crises in modern Europe. Millions of Ukrainians have been displaced and forced to seek safety in other countries, making the provision of adequate support and integration resources a priority across Europe. While the consequences of war are most severe among those directly exposed to violence, displacement itself introduces significant psychological burdens, including insecurity, grief, chronic stress, and uncertainty about the future (2, 3). These experiences highlight the importance of understanding factors that protect or undermine mental health in displaced populations.

Emerging evidence from studies conducted since 2022 indicates a substantial mental health burden among Ukrainians affected by the full-scale invasion, including those who fled to European host countries. Recent surveys and cohort studies have reported elevated rates of trauma-related symptoms as well as anxiety and depressive symptoms among Ukrainian refugees across Europe, with a sizeable proportion screening positive for probable PTSD and clinically relevant psychological distress (4–7). Acute stress reactions have also been documented in the early phase of displacement, highlighting the need for timely identification of vulnerable individuals (5).

These findings support the use of brief, standardized screening tools that capture both general psychological distress and trauma-related symptomatology in refugee populations. In this study, we therefore assessed distress using RHS-15 and HSCL-25, complemented by a checklist of DSM-5 trauma-related symptoms, and examined protective and adaptive processes through resilience and coping measures. This broader assessment framework is aligned with public health recommendations emphasizing both risk indicators and psychosocial resources in forced migration contexts (8).

Migration under conditions of forced displacement is closely tied to acculturation processes that may heighten perceived threat and stress, as individuals must navigate unfamiliar sociocultural norms, values, institutional structures, and—often—religious contexts. Challenges such as limited language proficiency, barriers in the labor market, and insufficient social support can intensify feelings of isolation, sadness, and loss associated with leaving one's previous life behind. These obstacles may negatively affect overall wellbeing and hinder effective adaptation to the host environment (9–11).

Importantly, although institutional and structural factors (e.g., welfare systems, access to healthcare, and legal protections) shape refugees' living conditions, the present study operationalizes psychological adaptation at the individual level, focusing on psychological distress, resilience, and coping strategies as measurable outcomes and potential protective factors in the context of forced displacement.

In this context, resilience has emerged as a key construct in understanding individual differences in psychological functioning during and after adversity. Although definitions vary, resilience generally refers to the capacity to maintain or regain psychological balance in the face of stress, using personal and environmental resources to support adaptation. It encompasses processes that help individuals minimize the negative impact of stressors, recover from adversity, and maintain relatively stable functioning. As such, resilience plays a crucial role in preventing more severe psychological difficulties and supporting positive adjustment among individuals exposed to trauma and displacement (10–13).

The diversity of national responses to the Ukrainian refugee crisis across Europe offers a unique opportunity for comparative research. Countries differ substantially in their welfare systems, migration policies, and integration infrastructures—from more centralized, state-led systems (e.g., Poland, Slovenia) to decentralized, NGO-driven models (e.g., Spain, Cyprus). Federal structures, such as in Germany, produce additional regional variation in access to services, whereas countries like Portugal combine inclusive policies with capacity limitations. Serbia, as a non-EU transit and temporary host country, operates largely outside EU protection mechanisms and relies more heavily on humanitarian support. These divergent institutional ecologies provide a valuable comparative lens for examining how societal contexts interact with individual psychological resources to shape refugees' mental health trajectories (14, 15). While these institutional differences are not directly measured in the present study, they provide the contextual backdrop for interpreting cross-national patterns. Accordingly, we focus on individual-level psychological distress, resilience, and coping strategies. In addition, we included internally displaced persons residing in Ukraine as a distinct analytical group to provide a reference point for individuals exposed to ongoing war-related stressors within the country of origin; Ukraine is therefore not treated as a host country but as a separate context of internal displacement. The aim of the study was to identify and compare cross-national differences in levels of psychological distress, resilience, and coping strategies among Ukrainian war refugees residing in seven European host countries and internally displaced persons in Ukraine.

## 2 Material and methods

### 2.1 Study design

This study used a cross-sectional, comparative survey design to identify and compare cross-national differences in levels of psychological distress, resilience, and coping strategies among Ukrainian refugees living in seven European host countries (Slovenia, Poland, Cyprus, Spain, Germany, Portugal, Serbia) as well as internally displaced persons residing in Ukraine. Internally displaced persons residing in Ukraine were treated as a distinct comparison group.

### 2.2 Participants and procedure

Participants were Ukrainian adults aged 18 years and older who had left Ukraine after the escalation of hostilities in February 2022 or who were internally displaced within Ukraine. Recruitment took place between March and June 2025 through refugee support organizations, Ukrainian community networks, NGOs, and social media platforms in each participating country. Respondents completed an online questionnaire available in Ukrainian, designed to ensure accessibility and linguistic accuracy. Participants residing in Ukraine represented internally displaced persons (IDPs) and were analyzed as a separate group rather than as part of the host-country comparisons.

Sampling strategy: a non-probability convenience sampling approach was used, supplemented by snowball recruitment (participants were encouraged to share the survey link within their networks) through refugee support organizations, Ukrainian community groups, and online platforms in each participating country.

A total of 1,631 respondents participated (Slovenia *n* = 104; Poland *n* = 715; Spain *n* = 92; Cyprus *n* = 94; Ukraine *n* = 232; Germany *n* = 101; Portugal *n* = 103; Serbia *n* = 190). The sample was predominantly female (78.6%), reflecting gender patterns in the displacement context (UNHCR 2025a). Participants' ages ranged from 18 to 65 years (*M* = 38.82, SD = 13.911). Marital status distribution was as follows: 55.6% (*n* = 906) were in a relationship and cohabiting with their partner, 16.4% (*n* = 267) were in a relationship with a partner residing in Ukraine, and 28.0% (*n* = 458) were unmarried. Most respondents were currently employed (67.8%), followed by students (13.5%), professionally inactive individuals (9.6%), retirees (6.0%), and those selecting “other” (3.1%). Detailed sociodemographic characteristics by country of residence (*n* and %) are presented in Table 1.

**Table 1:** Sociodemographic characteristics (*N* = 1,631).

**Measure**	**Category**	**Poland (*n* = 715)**	**Germany (*n* = 101)**	**Spain (*n* = 92)**	**Cyprus (*n* = 94)**	**Slovenia (*n* = 104)**	**Ukraine (*n* = 232)**	**Portugal (*n* = 103)**	**Serbia (*n* = 190)**
Gender	Male	171 (23.9%)	16 (15.8%)	17 (18.5%)	19 (20.2%)	20 (19.2%)	36 (15.5%)	15 (14.6%)	41 (21.6%)
	Female	540 (75.5%)	85 (84.2%)	71 (77.2%)	73 (77.7%)	81 (77.9%)	196 (84.5%)	87 (84.5%)	149 (78.4%)
	Non-binary	3 (0.4%)	0 (0.0%)	0 (0.0%)	2 (2.1%)	0 (0.00%)	0 (0.0%)	1 (1.0%)	0 (0.0%)
	Prefer not to say	1 (0.1%)	0 (0.0%)	4 (4.3%)	0 (0.0%)	3 (2.9%)	0 (0.0%)	0 (0.0%)	0 (0.0%)
Age	18–24	111 (15.5%)	12 (11.9%)	13 (14.1%)	6 (6.4%)	21 (20.2%)	126 (54.3%)	8 (7.8%)	9 (4.7%)
	25–34	195 (27.3%)	12 (11.9%)	17 (18.5%)	16 (17%)	22 (21.2%)	31 (13.4%)	27 (26.2%)	35 (18.4%)
	35–44	175 (24.5%)	37 (36.6%)	28 (30.4%)	31 (33%)	37 (35.6%)	50 (21.6%)	40 (38.8%)	47 (24.7%)
	45–54	141 (19.7%)	26 (25.7%)	13 (14.1%)	28 (29.8%)	19 (18.3%)	15 (6.5%)	19 (18.4%)	47 (24.7%)
	55–64	59 (8.3%)	9 (8.9%)	6 (6.5%)	7 (7.4%)	3 (2.9%)	1 (0.4%)	3 (2.9%)	33 (17.4%)
	65+	34 (4.8%)	5 (5.0%)	15 (16.3%)	6 (6.4%)	2 (1.9%)	9 (3.9%)	6 (5.8%)	19 (10%)
Marital status	In a relationship (my partner lives with me)	362 (50.6%)	43 (42.6%)	51 (55.4%)	69 (73.4%)	44 (42.3%)	212 (91.4%)	74 (71.8%)	51 (26.8%)
	In relationship (my partner lives in Ukraine)	98 (13.7%)	22 (21.8%)	21 (22.8%)	4 (4.3%)	22 (21.2%)	0 (0.0)	12 (11.7%)	68 (35.8%)
	Unmarried	254 (35.6%)	36 (35.6%)	20 (21.7%)	21 (22.3%)	38 (36.5%)	20 (8.6%)	17 (16.5%)	71 (37.4%)
Occupation	Student	70 (9.8%)	21 (20.8%)	12 (13%)	6 (6.4%)	36 (34.6%)	63 (27.2%)	7 (6.8%)	5 (2.6%)
	Employed physically	372 (52%)	27 (26.7%)	31 (33.7%)	24 (25.5%)	20 (19.2%)	36 (15.5%)	18 (7.5%)	32 (16.8%)
	Employed mentally	159 (22.2%)	19 (18.8%)	18 (19.6%)	44 (46.8%)	31 (29.8%)	133 (57.3%)	50 (48.5%)	92 (48.4%)
	Professionally inactive	56 (7.8%)	18 (17.8%)	10 (10.9%)	15 (16%)	13 (12.5%)	0 (0.0%)	21 (20.4%)	24 (12.6%)
	Retired	35 (4.9%)	8 (7.9%)	16 (17.4%)	2 (2.1%)	4 (3.8%)	0 (0.0%)	2 (1.9%)	31 (16.3%)
	Other	23 (3.2%)	8 (7.9%)	5 (5.4%)	3 (3.2%)	0 (0.0%)	0 (0.0%)	5 (4.9%)	6 (3.1%)

Values are presented as n (%).

Percentages are based on valid responses within each country.

This diverse sample reflects differences in both institutional regimes and migration trajectories, providing a unique basis for comparative analysis of psychological functioning.

### 2.3 Data collection and instruments

The study used standardized, validated psychometric instruments to ensure reliability and comparability across contexts. All instruments were administered in Ukrainian using the forward–backward translation procedure recommended by the World Health Organization to ensure linguistic and conceptual equivalence (16).

Where available, we used existing Ukrainian language versions of the measures (e.g., RHS-15 and COPE-60). For instruments without an available Ukrainian version (DSM-5 Trauma Symptoms Checklist, Resilience Scale-25), we conducted translation and cultural adaptation following the WHO recommendations.

Specifically, the DSM-5 Trauma Symptoms Checklist (owned by the Harvard Program in Refugee Trauma) and Resilience Scale-25 was translated into Ukrainian with the permission of the original authors. Two bilingual translators independently produced forward translations, which were then reconciled into a single version. The translation was reviewed by an expert panel that included mental-health professionals and Ukrainian-speaking co-authors affiliated with a Ukrainian university to ensure semantic and cultural equivalence. An independent bilingual translator, blinded to the original, performed a back-translation into English. Discrepancies were discussed and resolved by consensus, and the final version was pre-tested in a small pilot group of Ukrainian participants to confirm clarity and comprehensibility before data collection. Where required, permissions to use the psychometric instruments were obtained from the respective copyright holders, and we relied on the officially available Ukrainian-language versions for RHS-15 and COPE-60. For the DSM-5 Trauma Symptoms Checklist, the Ukrainian-language research version was created for use within this study only and was not distributed publicly; all intellectual property rights remain with the original owners.

The Refugee Health Screener (RHS-15) authored by David Hollifield was developed as a screening tool for the preliminary identification of mental health problems among individuals experiencing forced migration. The scale enables the detection of elevated risk of psychological distress and consists of 14 symptom-related items and one item assessing subjectively perceived distress. A positive screen is obtained when the sum of scores from the 14 symptom items is ≥12 or when subjective distress is rated at ≥5, indicating an increased risk of mental health problems. The RHS-15 has well-documented psychometric properties in refugee populations, demonstrating high criterion-related validity confirmed by its agreement with clinical interviews and tools such as the HSCL-25 and HTQ, as well as high sensitivity (approx. 0.81–0.95) with moderate specificity (approx. 0.70–0.85), making it an adequate screening tool for identifying individuals in a state of increased mental distress (17). For comparative analyses, mean item scores were calculated to ensure comparability with other symptom scales; higher values indicate greater psychological distress.

The DSM-5 Trauma Symptoms Checklist authored by American Psychiatric Association was used to assess exposure to traumatic events and the severity of post-traumatic symptoms, operationalized as continuous symptom indicators across four diagnostic clusters. The assessment encompasses somatic and affective dimensions, and symptom presence is analyzed quantitatively or qualitatively within four diagnostic clusters: intrusions, arousal, negative alterations in mood and cognition, and avoidance. It is most often used as a tool to support clinical assessment rather than as a fully standardized psychometric test (18).

The Hopkins Symptom Checklist (HSCL-25) by Leonard R. Derogatis was employed to measure symptoms of anxiety and depression using a four-point Likert scale, while also providing an index of overall psychological distress. The instrument consists of 25 items, of which 10 assess anxiety symptoms and 15 assess depressive symptoms. Score interpretation is based on the calculation of the arithmetic mean, with values ≥1.75 considered clinically significant; higher scores indicate greater symptom severity (19, 20).

Psychological resilience was measured using the Resilience Scale (RS-25). Scale was developed by Gail M. Wagnils and Heather M. Young. This instrument comprises 25 statements rated on a seven-point Likert scale and includes two main dimensions: personal competence and acceptance of self and life. In interpreting the results, scores ≤ 120 indicate low resilience, scores of 121–145 indicate moderate resilience, and scores ≥ 146 indicate high resilience (21).

Coping strategies were assessed using the COPE Inventory (COPE-60) by Charles S. Carver, Michael F. Scheier, Jagdish K. Weintraub, which consists of 60 items and encompasses 15 coping dimensions grouped into adaptive and maladaptive strategies. Scores are calculated separately for each coping strategy, allowing for the analysis of individual dimensions, in line with the assumptions of the present study. Higher scores indicate more frequent use of a given strategy (22, 23).

The three tools used in the study—HSCL-25, RS-25 and COPE-60—are characterized by good theoretical validity and cross-cultural validity confirmed in research, which justifies their use in the analyzed population (24–27).

### 2.4 Analytic strategy

Analyses were conducted using IBM SPSS Statistics and AMOS software. Data screening included checks for univariate and multivariate normality, multicollinearity, and missing data. Descriptive statistics (means, standard deviations) and bivariate Pearson correlations were calculated for all study variables to characterize the sample and examine zero-order associations between psychological distress, resilience, and coping strategies. To address the main research aim—namely, to identify and compare cross-national differences in levels of psychological distress, resilience, and coping strategies among Ukrainian war refugees— country of residence (seven host countries and Ukraine) was treated as a between-subjects factor. A series of one-way analyses of variance (ANOVAs) were conducted with host country as the independent variable and, respectively, psychological distress, resilience (RS-25 total score), adaptive coping, and maladaptive coping as dependent variables. Effect sizes were quantified using eta-squared (η^2^). Due to unequal sample sizes across countries, Games–Howell *post hoc* tests were applied for pairwise comparisons where omnibus effects were statistically significant.

In addition, preliminary one-way ANOVAs were conducted to examine the effects of selected sociodemographic variables (age group, gender, marital status, and employment status) on psychological distress (RHS-15 scores). Age was categorized into six groups (18–24, 25–34, 35–44, 45–54, 55–64, and 65+ years) to enable meaningful group comparisons and ensure adequate group sizes.

To further examine the independent contribution of sociodemographic variables to psychological distress, a univariate general linear model (GLM) was conducted with RHS-15 scores as the dependent variable and host country, gender, marital status, employment status, and age (continuous) entered simultaneously as predictors. This approach allowed for the assessment of cross-national differences while statistically controlling for relevant sociodemographic characteristics. Partial eta-squared (η^2^) was used as a measure of effect size. This GLM specification is equivalent to multiple linear regression with categorical predictors dummy-coded (country, gender, marital status, employment) and age entered as a continuous covariate.

Internal consistency was assessed using Cronbach's alpha. All instruments demonstrated good to excellent reliability: RHS-15 (α =0.923), HSCL-25 anxiety (α =0.897) and depression (α =0.936), DSM-5 total (α =0.957), RS-25 (α =0.935), adaptive coping (α =0.868), and maladaptive coping (α =0.761).

All tests were two-tailed, and statistical significance was set at *p* < 0.05.

### 2.5 Ethical considerations

The study was conducted in accordance with the Declaration of Helsinki and approved by the Bioethics Committee of Pomeranian Medical University in Szczecin, Poland (KB.006.41.2024). Participation was voluntary, and informed consent was obtained electronically before survey completion. Respondents received information about psychological support hotlines in each country. No personally identifiable data were collected.

## 3 Results

Preliminary analyses examined the effects of selected sociodemographic variables on psychological distress (RHS-15). One-way ANOVA results indicated significant main effects of age group (*F*_(5_, _1, 625)_ = 4.09, *p* = 0.001) and employment status (*F*_(5_, _1, 625)_ = 6.03, *p* < 0.001) on distress scores. In contrast, no significant effects were observed for gender (*F*_(3_, _1, 627)_ = 0.25, *p* = 0.860) or marital status (*F*_(2_, _1, 627)_ = 0.51, *p* = 0.600). These findings indicate that age and employment contribute to variability in psychological distress and were therefore considered in subsequent analyses.

To further examine the independent contribution of sociodemographic factors, a univariate general linear model was conducted including host country, gender, marital status, employment status, and age as predictors of psychological distress (RHS-15). After controlling for sociodemographic variables, country of residence remained a significant predictor of distress, *F*_(7_, _1, 611)_ = 38.79, *p* < 0.001, partial η^2^ = 0.144. Marital status (*p* = 0.003) and employment status (*p* = 0.004) also showed significant independent effects, whereas gender and age were not significant predictors in the adjusted model. The overall model explained 16.2% of the variance in psychological distress (R^2^ = 0.162).

A one-way ANOVA revealed statistically significant differences in psychological distress levels across the examined countries. These differences were observed for all assessed indicators—RHS-15, HSCL-25 (anxiety and depression), and the four DSM-5 trauma-related symptom clusters (intrusion, avoidance, negative alterations in cognition and mood, and arousal)—all *p* < 0.05.

Table 2 presents the mean scores obtained by participants in each host country, demonstrating clear variation in psychological burden. The highest levels of distress were reported in Slovenia, Ukraine, Portugal, Spain, and Germany, where mean RHS-15 scores and HSCL-25 and DSM-5 subscale scores were consistently elevated. For example, DSM-5 total scores in these countries ranged from 2.017 to 2.246, indicating more pronounced trauma-related symptoms.

**Table 2:** Means and standard deviations of psychological distress scores by country of residence.

**Measure**	**Poland**	**Germany**	**Spain**	**Cyprus**	**Slovenia**	**Ukraine**	**Portugal**	**Serbia**
RHS-15	1.293 (0.743)	1.838 (0.850)	1.825 (0.742)	0.760 (0.650)	1.847 (0.710)	1.848 (0.922)	1.774 (0.827)	1.140 (0.854)
HSCL-25 anxiety	0.668 (0.665)	0.923 (0.619)	1.195 (0.606)	0.423 (0.410)	1.147 (0.606)	1.100 (0.791)	1.141 (0.541)	0.694 (0.578)
HSCL-25 depression	0.704 (0.669)	1.105 (0.597)	1.357 (0.605)	0.355 (0.475)	1.187 (0.616)	1.194 (0.689)	1.237 (0.476)	0.719 (0.587)
DSM-5 intrusion	1.570 (0.635)	1.903 (0.729)	2.054 (0.581)	1.436 (0.753)	2.198 (0.736)	2.132 (0.788)	2.148 (0.666)	1.963 (0.674)
DSM-5 avoidance	1.829 (0.866)	2.074 (0.947)	1.940 (0.737)	1.410 (0.618)	2.255 (0.850)	2.009 (0.951)	2.049 (0.920)	1.879 (0.704)
DSM-5 neg. cog./mood	1.664 (0.654)	2.104 (0.711)	2.088 (0.581)	1.359 (0.522)	2.280 (0.722)	2.052 (0.703)	2.220 (0.648)	1.899 (0.627)
DSM-5 arousal	1.632 (0.644)	1.985 (0.714)	2.016 (0.622)	1.397 (0.587)	2.251 (0.673)	1.997 (0.779)	2.076 (0.676)	1.871 (0.617)
DSM-5 total	1.673 (0.628)	2.017 (0.702)	2.025 (0.555)	1.400 (0.567)	2.246 (0.633)	2.047 (0.740)	2.123 (0.617)	1.903 (0.590)

Values represent mean scores with standard deviations (SD) in parentheses.

One-way analyses of variance (ANOVA) were conducted with country of residence as the independent variable for each outcome variable.

Refugee health screener-15 (RHS-15).

Hopkins Symptom Checklist-25 (HSCL-25; anxiety and depression subscales).

DSM-5 trauma-related symptom clusters (intrusion, avoidance, negative alterations in cognition and mood, arousal) as well as DSM-5 total score.

Due to unequal sample sizes across countries.

Games–Howell post hoc tests were used for pairwise comparisons following significant omnibus effects. Statistical significance was set at p < 0.05 (two-tailed).

Statistical significance was set at p < 0.05 (two-tailed).

RHS-15, refugee health screener-15;

HSCL-25, Hopkins symptom checklist-25;

DSM-5, diagnostic and statistical manual of mental disorders.

In contrast, the lowest distress levels were observed in Cyprus and Poland, where mean scores on all measures were markedly lower. RHS-15 scores in these countries were 0.760 and 1.293, respectively, and DSM-5 total scores were 1.400 and 1.673, reflecting substantially reduced symptom severity compared with higher-distress countries.

Games–Howell *post hoc* tests indicated that distress levels in Cyprus were significantly lower than in most other countries, and that Poland also differed significantly from several higher-distress country groups (*p* < 0.05). Overall, significant cross-national differences in psychological distress were observed across host countries (Table 2).

A one-way ANOVA revealed significant differences in resilience levels across host countries (*F* = 22.01, *p* < 0.001). Table 3 presents the country-level means, standard deviations, and confidence intervals for the RS-25 resilience scores. The results demonstrate marked variation in resilience among Ukrainian refugees residing in different national contexts (η^2^ = 0.087).

**Table 3:** Resilience (RS-25) scores by country of residence.

**Country**	***n***	**Mean**	**SD**	**SE**	**95% CI (Lower)**	**95% CI (Upper)**
Poland	715	5.417	1.105	0.041	5.335	5.498
Germany	101	5.447	0.982	0.098	5.253	5.641
Spain	92	4.409	1.003	0.105	4.202	4.617
Cyprus	94	5.111	1.006	0.104	4.905	5.317
Slovenia	104	4.778	0.874	0.086	4.608	4.948
Ukraine	232	4.733	1.276	0.084	4.568	4.898
Portugal	103	4.724	1.262	0.124	4.478	4.971
Serbia	190	5.192	0.932	0.068	5.059	5.325
*Total*	1,631	*5.136*	*1.140*	*0.028*	*5.081*	*5.192*

Values represent mean scores and standard deviations (SD) for the Resilience Scale (RS-25; 25-item version; higher scores indicate higher resilience).

SE, standard error;

CI, confidence interval.

A one-way analysis of variance (ANOVA) tested country differences in resilience.

Due to unequal sample sizes, Games–Howell post hoc tests were used for pairwise comparisons following a significant omnibus effect.

Statistical significance was set at p < 0.05 (two-tailed).

The highest resilience levels were observed in Poland (*M* = 5.417) and Germany (*M* = 5.447), where participants reported the strongest perceived personal competence and acceptance of self and life. Confidence intervals for both countries (Poland: 5.335–5.498; Germany: 5.253–5.641) were narrowly distributed, indicating relatively stable and elevated resilience scores.

In contrast, the lowest resilience levels were found in Spain (*M* = 4.409, 95% CI = 4.202–4.617), where scores were consistently lower than in all other host countries. Participants in Slovenia (*M* = 4.778), Ukraine (*M* = 4.733), and Portugal (*M* = 4.724) reported intermediate resilience levels, reflecting moderate perceived adaptive capacity. Cyprus (*M* = 5.111) and Serbia (*M* = 5.192) also fell within the mid-range but showed relatively higher means within this group.

Overall, the significant ANOVA results and observed mean differences indicate substantial cross-national variation in resilience. These findings indicate significant cross-national differences in resilience among Ukrainian refugees.

Analyses revealed significant cross-national differences in both adaptive coping (*F*_(7_, _1, 622)_ = 25.85, *p* < 0.001) and maladaptive coping (*F*_(7_, _1, 623)_ = 24.85, *p* < 0.001) (η^2^_adaptive = 0.100; η^2^_maladaptive = 0.097). Games–Howell *post hoc* tests were applied to identify significant pairwise differences due to unequal group sizes. Mean scores and standard deviations for each country are presented in Table 4. The highest levels of adaptive coping were reported in Poland (*M* = 2.386), Slovenia (*M* = 2.359), and Germany (M = 2.402), indicating a relatively strong engagement in constructive strategies such as active problem-solving and positive reframing. These countries also demonstrated low variability, suggesting stable patterns of adaptive coping among respondents.

**Table 4:** Adaptive and maladaptive coping scores by country of residence.

**Country**	***n***	**Adaptive coping (mean)**	**Adaptive SD**	**Maladaptive coping (mean)**	**Maladaptive SD**
Poland	715	2.386	0.434	2.260	0.362
Germany	101	2.402	0.547	2.336	0.509
Spain	92	2.167	0.405	2.119	0.346
Cyprus	94	1.801	0.660	1.752	0.596
Slovenia	104	2.359	0.283	2.299	0.275
Ukraine	232	2.282	0.344	2.191	0.287
Portugal	103	2.256	0.568	2.174	0.526
Serbia	190	2.166	0.376	2.130	0.354

Values represent mean scores and standard deviations (SD) for adaptive and maladaptive coping as assessed with the COPE Inventory (COPE-60; 60-item version).

Higher scores indicate more frequent use of the coping strategy.

One-way analysis of variance (ANOVA) tested country differences in adaptive and maladaptive coping.

Adaptive coping and maladaptive coping represent aggregated subscales of the COPE Inventory.

Due to unequal sample sizes across countries, Games–Howell post hoc tests were used for pairwise comparisons following significant omnibus effects.

Statistical significance was set at p < 0.05 (two-tailed).

Conversely, the lowest adaptive coping scores were observed in Cyprus (*M* = 1.801) and Serbia (*M* = 2.166), indicating reduced reliance on adaptive approaches within these contexts. Spain (*M* = 2.167), Portugal (*M* = 2.256), and Ukraine (*M* = 2.282) fell into intermediate ranges, suggesting moderate use of adaptive coping strategies.

Maladaptive coping showed a similar but less pronounced pattern of cross-national variation. Although differences were statistically significant, mean scores clustered more closely together across countries. Poland, Slovenia, and Germany exhibited slightly higher maladaptive coping tendencies compared with Cyprus and Serbia; however, the differences were smaller relative to those observed for adaptive coping. These results indicate that adaptive coping strategies vary more strongly across countries than maladaptive coping strategies.

## 4 Discussion

The present study demonstrates clear and statistically significant cross-national differences in psychological distress, resilience, and coping strategies among Ukrainian refugees, underscoring the central role of host-country contexts in shaping mental health outcomes during forced displacement. From a public health perspective, these findings confirm that refugee mental health is a population-level issue influenced by structural, institutional, and policy determinants rather than solely by individual exposure to trauma.

Marked cross-national differences in psychological distress were observed. Ukrainian refugees residing in Cyprus and Poland reported notably lower distress, whereas those in Slovenia, Ukraine, Spain, Germany, and Portugal exhibited higher symptom burdens. These patterns are consistent with socio-ecological models of adaptation, which emphasize that mental health outcomes are embedded within broader institutional, cultural, and political systems (13, 28). However, because institutional and political factors were not directly assessed in the present study, these interpretations should be considered contextual and hypothesis-generating rather than causal explanations of the observed differences (29). Importantly, contemporary public health frameworks developed by the World Health Organization and UNHCR similarly conceptualize refugee mental health as shaped by post-migration conditions, access to services, and system-level protection mechanisms, rather than by trauma exposure alone (30).

At the same time, cross-national differences may partly reflect compositional differences between the country samples (e.g., age distribution, gender balance, and employment status), rather than the country context alone. To address this possibility, additional analyses were conducted including sociodemographic variables as covariates. Host country remained a significant predictor of psychological distress after controlling for age, gender, marital status, and employment, indicating that cross-national differences cannot be explained solely by compositional characteristics of the samples. Nevertheless, future studies should further investigate these relationships using multilevel or longitudinal designs to disentangle contextual and individual-level mechanisms more precisely.

Resilience also differed substantially across national contexts, with the highest levels observed in Poland and Germany and the lowest in Spain and Slovenia. Importantly, resilience does not imply the absence of psychological distress; rather, protective resources and symptom burden may co-occur, especially under ongoing adversity (31, 32). From a public health standpoint, resilience should be understood not merely as an individual psychological trait but as an outcome influenced by structural determinants such as legal stability, access to healthcare, labor market inclusion, and social integration. Previous research shows that, beyond exposure to war-related violence, mental health outcomes among refugees are strongly shaped by post-migration stressors, including family separation, financial difficulties, problems with employment or underemployment, discrimination, and limited access to healthcare, all of which have been consistently associated with poorer mental health and increased psychological distress (33). The higher resilience observed in Poland and Germany may therefore reflect the combined effects of cultural proximity, established Ukrainian diasporas, and comparatively well-developed institutional support infrastructures.

This conceptualization helps explain seemingly paradoxical patterns such as the co-occurrence of elevated distress and relatively high resilience in some contexts (e.g., Germany). In line with the two-continua perspective on mental health, positive psychological resources and mental health symptoms represent related but distinct dimensions; individuals may therefore report substantial distress while simultaneously maintaining resilience-related capacities for adaptation and functioning (32). For refugees, this may reflect simultaneous exposure to persistent post-migration stressors (e.g., uncertainty, bureaucratic demands, and separation from family) alongside access to supportive resources and community networks that sustain resilience (29).

Cross-national variation in coping strategies further reinforces the importance of post-migration environments for public mental health. Refugees residing in more structured and coordinated reception systems, such as Poland, Germany, and Slovenia, reported higher levels of adaptive coping, whereas lower levels were observed in Cyprus and Serbia. Previous research demonstrates that adaptive coping among refugees is closely linked to integration-related conditions, including access to employment, education, and psychosocial services (21, 28). In line with WHO and UNHCR guidelines on mental health and psychosocial support (MHPSS), these findings suggest that strengthening everyday functioning and social participation may be as important as clinical interventions for promoting adaptive coping in displaced populations (30).

A closer examination of these cross-national patterns highlights the decisive role of institutional ecologies in shaping psychological adaptation. Lower distress levels in Poland and Cyprus appear consistent with reception systems characterized by greater predictability and clearer administrative pathways. In contrast, higher distress in Spain and Slovenia coincides with fragmented reception structures, administrative delays, and limited psychosocial capacity. Elevated distress in Germany and Portugal may reflect, respectively, the heterogeneity of federal reception frameworks and capacity constraints within welfare-oriented systems. Serbia, operating largely outside the EU protection regime and relying primarily on humanitarian rather than integration-oriented provision, exhibited moderate distress alongside comparatively lower adaptive coping resources (34). These findings align with comparative analyses showing that restrictive or unstable integration policies are associated with poorer mental health outcomes among refugees and migrants (35).

More broadly, the results are consistent with European public health evidence documenting persistent inequalities in access to healthcare and mental health services among refugee populations. Recent reports from Eurofound and the European Center for Disease Prevention and Control highlight substantial cross-national disparities in healthcare access, service continuity, and mental health provision for refugees within the European Union, particularly during periods of rapid displacement and system overload (36). Such inequalities likely contribute to the observed variation in distress, resilience, and coping across host countries.

Taken together, these findings demonstrate that institutional architectures are not passive contexts but active determinants of mental health trajectories among forcibly displaced populations. From a public health perspective, strengthening reception systems, reducing administrative barriers, and ensuring equitable access to mental health and social services are critical for mitigating long-term psychological distress and supporting resilience at the population level.

Given the broad age range of the participants (18–65 years), age-related differences in psychological distress, resilience, and coping may have influenced the observed cross-national patterns; however, age did not remain a significant independent predictor of psychological distress after adjustment for other sociodemographic variables in the multivariable model (37–39). For example, older adults may differ from younger adults in both exposure to cumulative stressors and preferred coping strategies. Although the present study focused on country-level comparisons, age-related mechanisms may still interact with contextual factors, even though age was not an independent predictor in the adjusted analyses. Future research should therefore examine potential age-by-country interactions and longitudinal age effects to clarify whether age moderates psychological adaptation across different institutional settings.

Future research should extend cross-national analyses to additional host countries and apply longitudinal designs to examine how changes in institutional arrangements influence mental health trajectories over time. Such evidence is essential for informing public health strategies and policy reforms aimed at promoting sustainable psychological adaptation during prolonged displacement.

### 4.1 Strengths and limitations

This study has several strengths. It provides one of the largest cross-national comparisons of Ukrainian war refugees across multiple European host countries, complemented by a distinct group of internally displaced persons residing in Ukraine. The use of validated measures and a common survey protocol enabled harmonized assessment of psychological distress, resilience, and coping across diverse settings. Moreover, focusing on multiple psychological constructs offers a more comprehensive picture of adaptation than studies limited to distress outcomes alone.

Several limitations should also be acknowledged. The cross-sectional design precludes causal inference and does not capture changes in mental health over time. Recruitment relied on non-probability sampling (convenience and snowball sampling), which may limit representativeness and introduce self-selection bias. In addition, sample sizes differed substantially between countries, and we did not directly measure institutional or policy-level factors that could help explain cross-national differences. Moreover, we did not directly assess refugees' post-migration experiences in the host countries (e.g., post-migration living difficulties, perceived discrimination, language barriers, or access to services), which limits our ability to empirically test mechanisms underlying the observed cross-national differences. In addition, educational level was not assessed in the present study, which limits the possibility of examining its potential association with psychological distress and cross-national differences. Although sociodemographic variables were included in additional multivariable analyses, residual confounding cannot be entirely excluded.

## 5 Conclusions

This study demonstrates substantial cross-national differences in psychological distress, resilience, and coping strategies among Ukrainian refugees across European host countries and internally displaced persons in Ukraine, highlighting the importance of a public health perspective on forced displacement.

The findings underscore the potential importance of institutional, social, and policy environments in shaping psychological adaptation during forced displacement. Although institutional and policy environments were not directly measured, host country remained a significant predictor of psychological distress after adjustment for key sociodemographic variables, suggesting that cross-national differences are unlikely to be explained solely by sample composition. Future public health research should explicitly integrate system-level indicators to empirically test these contextual influences.

## Data Availability

The raw data supporting the conclusions of this article will be made available by the authors, without undue reservation.
